# Establishment of a striped catfish skin explant model for studying the skin response in *Aeromonas hydrophila* infections

**DOI:** 10.1038/s41598-021-98583-8

**Published:** 2021-09-24

**Authors:** Ru-Fang Siao, Chia-Hsuan Lin, Li-Hsuan Chen, Liang-Chun Wang

**Affiliations:** Department of Marine Biotechnology and Resources, National Sun Yat-Sen University, Kaohsiung, Taiwan

**Keywords:** Ichthyology, Skin models, Bacterial host response, Marine microbiology

## Abstract

Teleost fish skin serves as the first line of defense against pathogens. The interaction between pathogen and host skin determines the infection outcome. However, the mechanism(s) that modulate infection remain largely unknown. A proper tissue culture model that is easier to handle but can quantitatively and qualitatively monitor infection progress may shed some lights. Here, we use striped catfish (*Pangasius hypophthalmus*) to establish an ex vivo skin explant tissue culture model to explore host pathogen interactions. The skin explant model resembles in vivo skin in tissue morphology, integrity, and immune functionality. Inoculation of aquatic pathogen *Aeromonas hydrophila* in this model induces epidermal exfoliation along with epithelial cell dissociation and inflammation. We conclude that this ex vivo skin explant model could serve as a teleost skin infection model for monitoring pathogenesis under various infection conditions. The model can also potentially be translated into a platform to study prevention and treatment of aquatic infection on the skin in aquaculture applications.

## Introduction

Fish skin is the first barrier to infection responding to environmental changes and aquatic pathogens^1^. The skin of scaleless teleost fish, such as catfish and eels, are of economic importance and is composed of the epidermis and dermis. The epidermis is the outermost region and ranges from two to multiple cell layers, determined by species, age, and anatomical location^2^. The epidermis contains various types of cells, including stratified epithelial and specialized mucous-secreting cells. The underlying dermis is composed of fibroblast cells that secret collagen, making up the soft connective tissue layer. When encountering pathogens, the skin can respond in several ways. Cytokine production can signal and attract neutrophils, mast cells, and macrophages for pathogen clearance^3^. Mucous is able to prevent pathogen attachment by sloughing off^4^. Antimicrobial peptides, lectins, lysozyme, and proteases secreted from epithelial cells located in the fish epidermis have been shown to kill pathogens by various mechanisms^5^. The cytokine response, relying on signal transduction pathways from pathogen recognition receptors such as Toll-like receptors (TLRs) to induce cytokine expression such as Interleukins (ILs), Tumor necrosis factor (TNF), and Interferons (IFNs) plays an essential role in pathogen clearance as part of the innate immune response^6^. These secretions, along with cells from the epidermis, are essential for responding to pathogen invasion. Nevertheless, little is known of the mechanisms by which pathogens initiate and produce infection on the host skin.

Current studies of the host–pathogen interaction mechanisms rely on observing the fish in vivo^7^ or using single epithelial^8^ and immune cell culture systems^9^. The majority of host–pathogen interaction studies use the fish in vivo to observe survival rate, physiological change, and the presence of pathogenic bacteria in specific organs or tissues to deduce infection routes and mechanisms. However, the in vivo model has its limitations in detailing the progression of the infection. First, host gene expression can be different in each experimental individual. Oleksiak et al. has shown that the gene expression can significantly differ within the same natural population of teleost fish^10^. A similar study of rainbow trout has further demonstrated differences in protein synthesis and turnover between the same teleost breed^11^. Second, differences exist in the associated microbiome from fish to fish. The microbiome is defined as the microbial community with distinct physio-chemical properties^12^. However, it was found that within the same species of fish, one can find different mucosal microbiomes, potentially impacting result interpretation and reproducibility^13^. Lastly, the teleost infection in vivo undergoes a complicated process containing interactions among the environment, pathogen, host, and microbiome. Using in vivo models to detail the infection mechanism may overlook these interactions leading to ambiguous conclusions^14^. For practical purposes, the number of fish needed to be sacrificed for data validity can be enormous. Therefore, an alternative model may be considered.

Primary and cell line-based epithelial or immune cells were considered alternative models for studying fish skin infection because of their simple, stable, and easy-to-handle properties. However, using epithelium cells or immune cells alone to study mechanisms is a double-edged sword due to the limited interpretation from single-cell culture. Despite the convenience of manipulation, it has been generally recognized that single-cell culture does not reflect the in vivo cellular processes due to the lack of multicellular interactions^15^. Nevertheless, teleost fish cell lines or primary cell culture is still the only choice besides the teleost fish in vivo experiments. To date, there are many cell lines that have been widely used, with the gill cell lines the most widely used. Even though epithelial polarity was not fully explored, the cultured RTgill-W1 and G1B cell lines on a semipermeable insert are able to form a multilayered epithelium for parasite infection study^8,16^. The primary fish gill system has then been studied and established for aquatic environmental monitoring^17^. Nonetheless, in both cell line and primary cell culture, gene expression and protein function may significantly differ from in vivo models due to the lack of multiple cell type signaling. Mouse and rat model have demonstrated these differences, though it has not been intensively examined in teleost fish^18,19^. As a result, a novel model between in vitro and in vivo with all the skin cell varieties present and integrated is potentially more representative. One study that resembles the new model proposed above established the everted intestinal sac culture using the whole catfish intestine to examine substance absorption in the digestive system^20^. Another study used the medaka scaled skin mounted on coverslips as epidermal sheets to examine tissue repair^21^. An ideal fish skin model would exhibit epithelial polarity and be able to mimic osmoregulation and barrier function. A previous study showed that an ex vivo human peritoneal tissue model, culturing internal and external surfaces separately, can be established with epithelium polarity for exploring tumor-peritoneal interactions^22^. A recent study has also used toad skin explant ex vivo to successfully monitor viral infection from external to the internal surface of the skin^23^. Nonetheless, a similar study in teleost fish skin has not been explored. Therefore, developing an ex vivo skin model system comparable to the models above can potentially mimic fish mucosal skin and serve as an alternative model for infection research.

In aquaculture, *Aeromonas hydrophila* (AH) is an important freshwater pathogen and can induce infection-associated death. Historically, AH has been suggested as an important pathogen in catfish farming systems, responsible for cutaneous ulceration and muscle necrosis. It may also cause aeromonad septicemia by hypervirulent strains^24^. In 2017, AH infections caused the loss of 3 million pounds of farm-raised catfish in Alabama, USA, making AH the primary pathogen to catfish species (Hemstreet, AL Fish Farming Center). This study aimed to establish an ex vivo mucosal skin model from *Pangasius hypophthalmus*, an economical catfish species in Southeast Asia, for better studying *Aeromonas hydrophila* infection^25^. The ex vivo skin model can mimic in vivo fish skin tissue by presenting most skin properties, including epidermis with epithelial cells and dermis with dense connective tissue. Due to its organ-based property, it is practically suitable for infection study as well as physiological study. Several skin samples can be processed from a single fish, thus, reducing experimental error from genetic differences of the fish. Moreover, the need for labor, space, and facilities can be significantly reduced due to the decreased fish number used in experiments. Eventually, this model can serve as a multi-purpose platform for fish mucosal surface research and an alternative or additive approach besides the fish in vivo.

## Results

### The *P. hypophthalmus* skin model preserves tissue morphology and integrity

**Figure 1:**
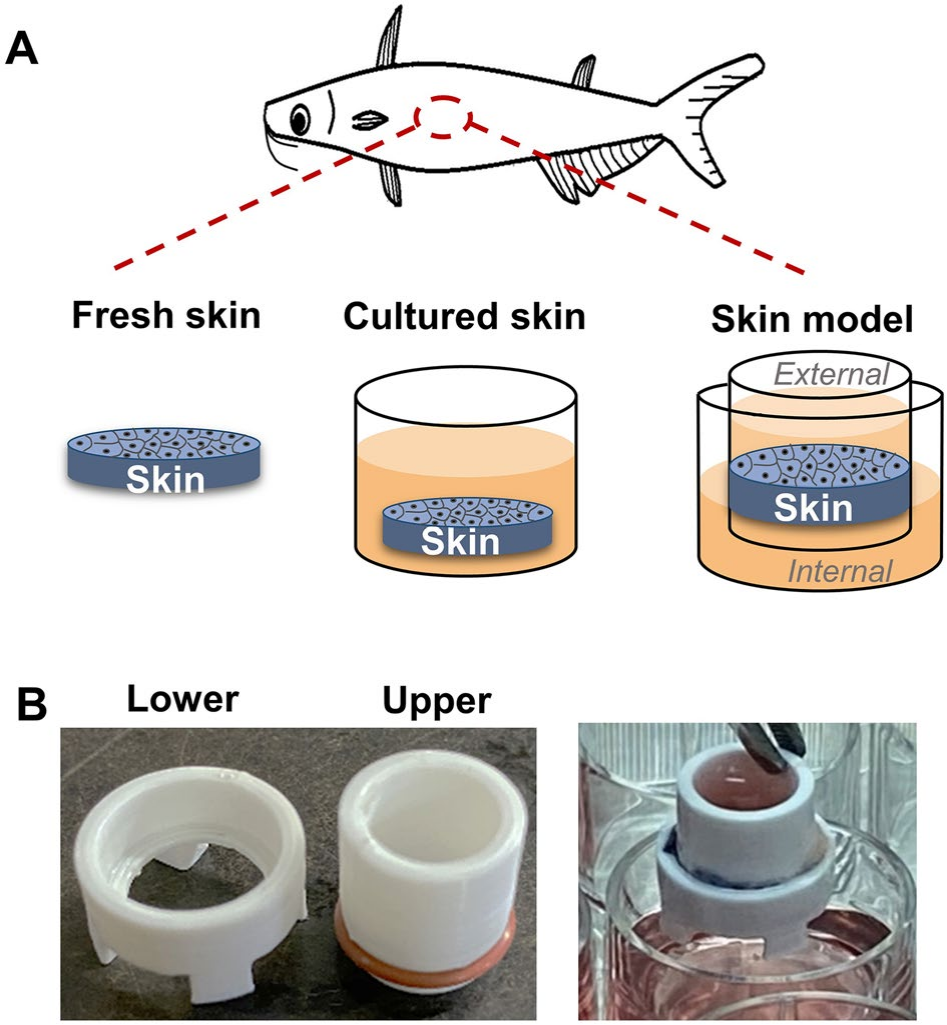
The illustration of the fresh skin, the cultured skin, and the skin model in this study. (**A**) The skin removed from fish was directly used as fresh skin (left), cultured in the media as cultured skin (middle), or media-cultured with external- and internal-facing region separated as the skin model (right). (**B**) Images of the upper and lower crowns (left) and the mounted crowns ready for the skin model culture (right).

**Figure 2:**
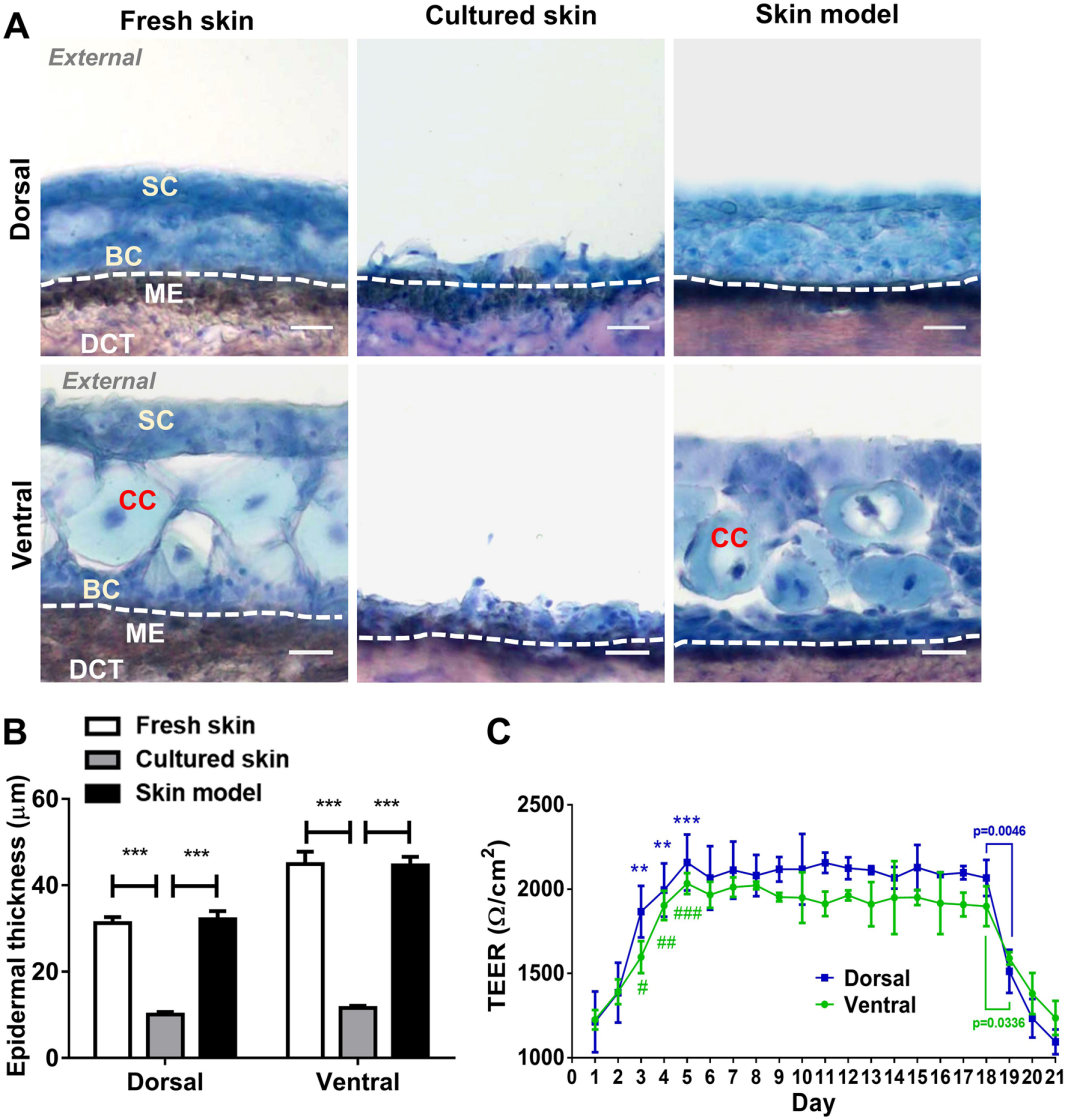
Morphological and functional examination of the skin model. Fresh skin and 5-day cultured skin and skin model were cryo-preserved and sectioned. (**A**) Tissue sections of dorsal and ventral regions were stained with Giemsa stain. Sections showing the superficial epithelial cells (SC), basal epithelial cells (BC), melanophores (ME), dense collagenous tissue (DCT), club cells (CC). Shown are representative images, Bar = 10 μm. (**B**) Images used to determine epidermal thickness. All data are shown as mean values (± SEM). Four fish with three replicates of tissue from each were used. The morphometric evaluation was determined from 20 randomly selected fields per sample in each condition. Statistical significance was determined using one-way ANOVA followed by Bonferroni post hoc test for comparison of epidermal thickness between three conditions under the same subject numbers. (**C**) The transepithelial electrical resistance (TEER) was measured in the skin model for a consecutive 21 days. All data were shown as mean values (± SEM). Four fish and three replicates of tissue from each were used. Statistical significance was determined by using Student’s t-test. ***p ≤ 0.001; **p ≤ 0.01; *p ≤ 0.05 versus Dorsal group Day1; ^###^p ≤ 0.001; ^##^p ≤ 0.01; ^#^p ≤ 0.05 versus Ventral group Day1.

Skin was removed and trimmed from the striped catfish and used as fresh skin, media-cultured (cultured skin), or media-cultured by separating external- and internal-facing region as the skin model (Fig. 1A), using a specially fabricated device as described in (Fig. 1B). This system is analogous to a traditional transwell system except that the fish skin doubles as the transwell membrane. To examine if the skin tissue integrity was maintained in the skin model, tissue sections of fresh skin, cultured skin, and skin model of the dorsal or ventral regions were stained with Giemsa solution. The tissue morphology and integrity of the skin were then examined by light microscopy (Fig. 2A). We found that the skin model, but not cultured skin, resembles fresh skin in both regions. The skin model and fresh skin of the ventral region showed a bottom layer of basal epithelial cells (BC) and superficial epithelial cells (SC) with club cells (CC) in between, whereas cultured skin only expressed a thin layer of basal epithelial cells. To determine if the epidermal layer was sustained during culture, the epidermal thickness of fresh skin, cultured skin, and skin model was measured and compared (Fig. 2B). We found that the thickness of the skin model remained unchanged while the cultured skin was 65% decreased in dorsal and 74% decreased in ventral compared to fresh skin. The transepithelial electrical resistance (TEER) is the measurement of electrical resistance across the epithelial layer and can be applied to quantify the barrier integrity during their stages of growth^26^. We used this measurement to examine the integrity of the epithelial layer in the skin model and monitored for consecutive 21 days (Fig. 2C). The TEER of the skin model showed a significant increase from day 1 to day 5 at the maximum of 2158 Ω/cm^2^ in dorsal and 2034 Ω/cm^2^ in ventral on day 5 and were maintained till day 18. A significant decrease of TEER was found from day 19 to 21 until the TEER returned to the level of day 1. These data show that the overall tissue morphology and cell integrity of the dorsal or ventral skin model resembled the fresh skin for a period of ~ 14 days.

### Tight junction components are expressed in the epidermis of the skin model

**Figure 3:**
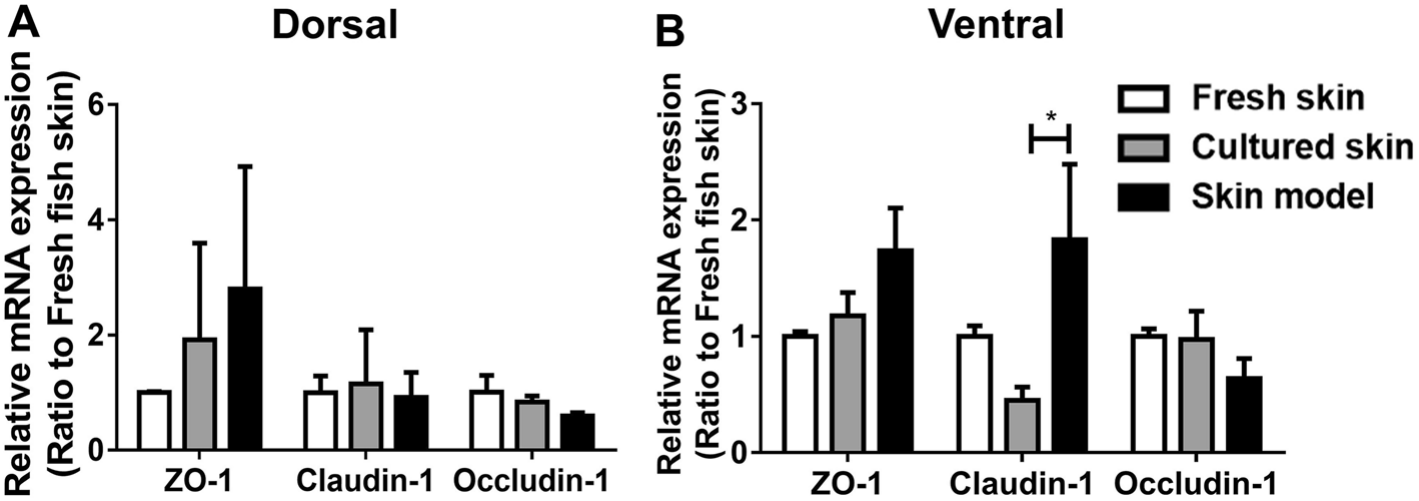
The mRNA expression level of tight junction components in the skin model. The mRNA expression level of ZO-1, Claudin-1 and Occludin-1 in cultured skin and skin model was relative to the expression of the fresh skin assigned a value of 1 [transcript abundance for each gene was normalized using housekeeping gene elongation factor 1-alpha (EF1-α)]. All data were shown as mean values (± SEM). Four fish and two replicates of tissue were used. Statistical significance was determined by using one-way ANOVA followed by Bonferroni post hoc test for comparison of expression between three conditions. *p ≤ 0.05.

Previous studies have shown that tight junction, as part of epithelial intercellular adhesion complex, plays an essential role in the teleost fish skin barrier function and osmolality regulation^27–29^. Lack or changes of junction expression level can ultimately lead to the increase in the epidermal permeability^29^, resulting in vulnerability to environmental pathogens. Since TEER measurement showed growth and maintenance of barrier integrity in our skin model, we then examined the expression level of tight junction components in the skin model. To determine if the skin model maintains the same mRNA expression levels of tight junction components as the fresh skin, Claudin-1, Occludin-1, and ZO-1, were examined using RT-qPCR (Fig. 3). In dorsal regions, the expression level of Claudin-1, Occludin-1, and ZO-1 were not significantly altered in cultured skin and the skin model compared to fresh skin (Fig. 3A). In the ventral region, the expression of Claudin-1 in the skin model had a threefold increase than cultured skin, while little alteration of Claudin-1, Occludin-1, and ZO-1 was observed compared to fresh skin (Fig. 3B). These data suggest expression level of the tight junction components in the skin model should resemble the fresh skin; therefore, the skin model can be used to study the skin barrier function.

### The skin model preserves the distribution and function of goblet cells

**Figure 4:**
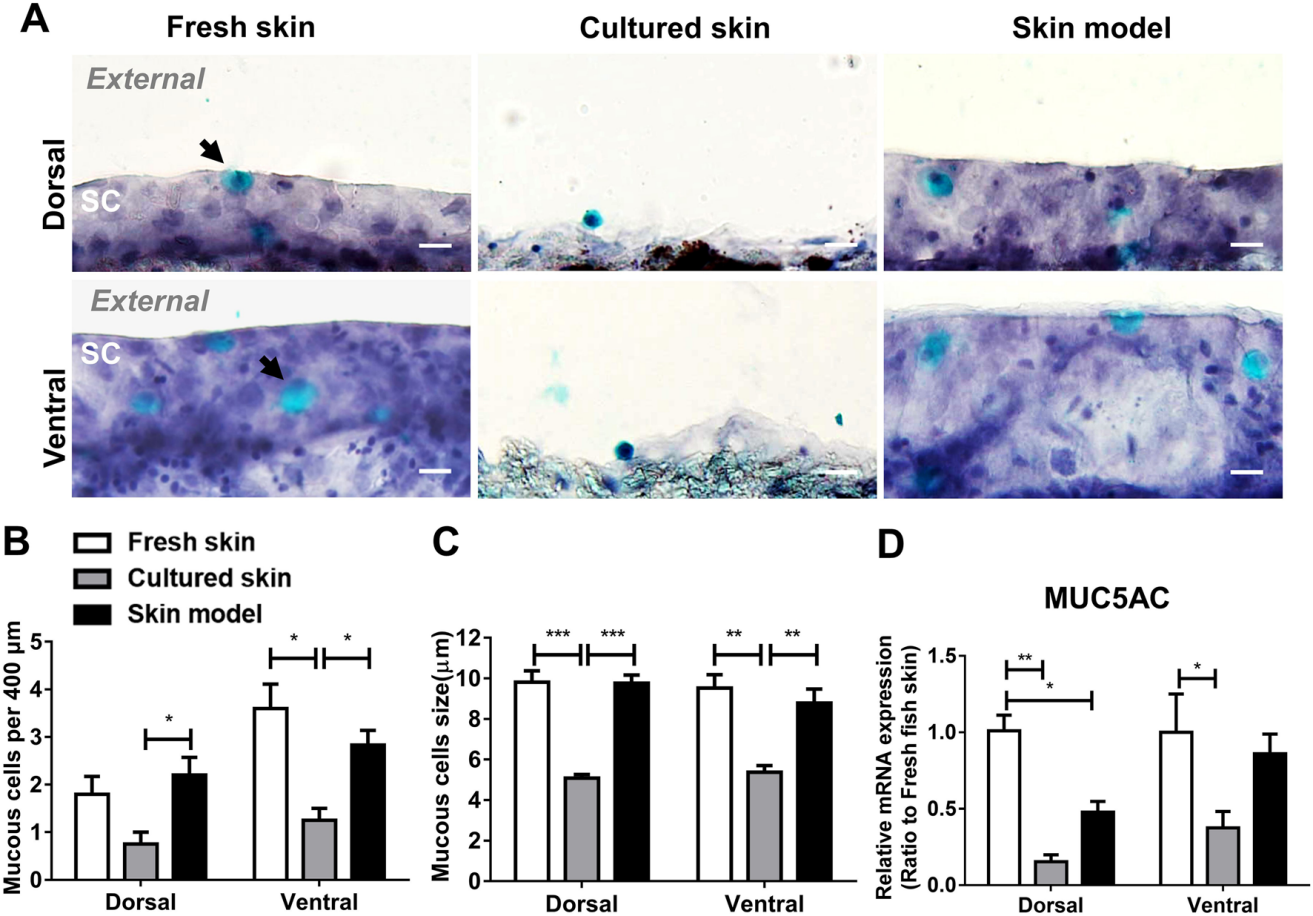
Morphometric analysis and mucin expression of goblet cell in the skin model. Samples from fresh skin, 5-day cultured skin, and skin model were cryo-preserved and sectioned. (**A**) Tissue sections of dorsal and ventral regions skin were stained with Alcian blue-hematoxylin stain. Arrows point the goblet cells located at superficial epithelial cell (SC) surface. Shown are representative images, Bar = 10 μm. Images were used to determine (**B**) the number and (**C**) average size of goblet cells. All data were shown as mean values (± SEM). Four fish and three replicates of tissue were used. The morphometric evaluation was determined from 20 randomly selected fields per sample in each condition. (**D**) MUC5AC expression was measured by RT-qPCR and compared relative to the expression in fresh skin. All data were shown as mean values (± SEM). Four fish and three replicates of tissue were used. Statistical significance was determined by using one-way ANOVA followed by Bonferroni post hoc test for comparison of expression between three conditions. ***p ≤ 0.001; **p ≤ 0.01; *p ≤ 0.05.

Fish skin mucous and various antimicrobial secretion acts as another layer of barrier against pathogens^4^. The main component, gel-forming mucous, is secreted mainly by goblet cells (GC)^30^. The maintenance of GC number and mucous secretion would indicate the status of healthy skin. To determine if the skin model can preserve GCs distribution within the epidermis similar to the fresh skin, the skin model was stained with Alcian blue-hematoxylin stain, imaged, and quantified for subsequent morphometric analysis. In both dorsal and ventral regions, the GCs were distributed on the outmost layer of epithelial cells and in the epidermis in both skin model and fresh skin, while sporadic GCs were distributed on the single layer of the epidermis in cultured skin (Fig. 4A). In both dorsal and ventral skin, the number and size of GCs in the skin model resembled fresh skin, whereas a significant decrease in both metrics found in cultured skin (Fig. 4B,C). We investigated the activity of GCs by examining the expression level of MUC5AC, a gene for mucin that is responsible for gel-forming mucous secretion (Fig. 4D). In the dorsal region, 55% and 88% expression decrease in MUC5AC was observed in the skin model and cultured skin compared to fresh skin. In the ventral region, no significant change was observed between the skin model and fresh skin (P = 0.336) but a 60% expression decrease in the cultured skin. In addition, mucous secreted from GC were observed in both fresh skin and the skin model located at the superficial epithelial cell surface while no secretion was observed in the cultured skin (Supplementary Fig. S1).These data suggest that goblet cells in the skin model are preserved in number and function and can represent fresh skin fully in the ventral and partially in the dorsal region.

### The skin model expresses different levels of innate immune factors compared with fresh skin

**Figure 5:**
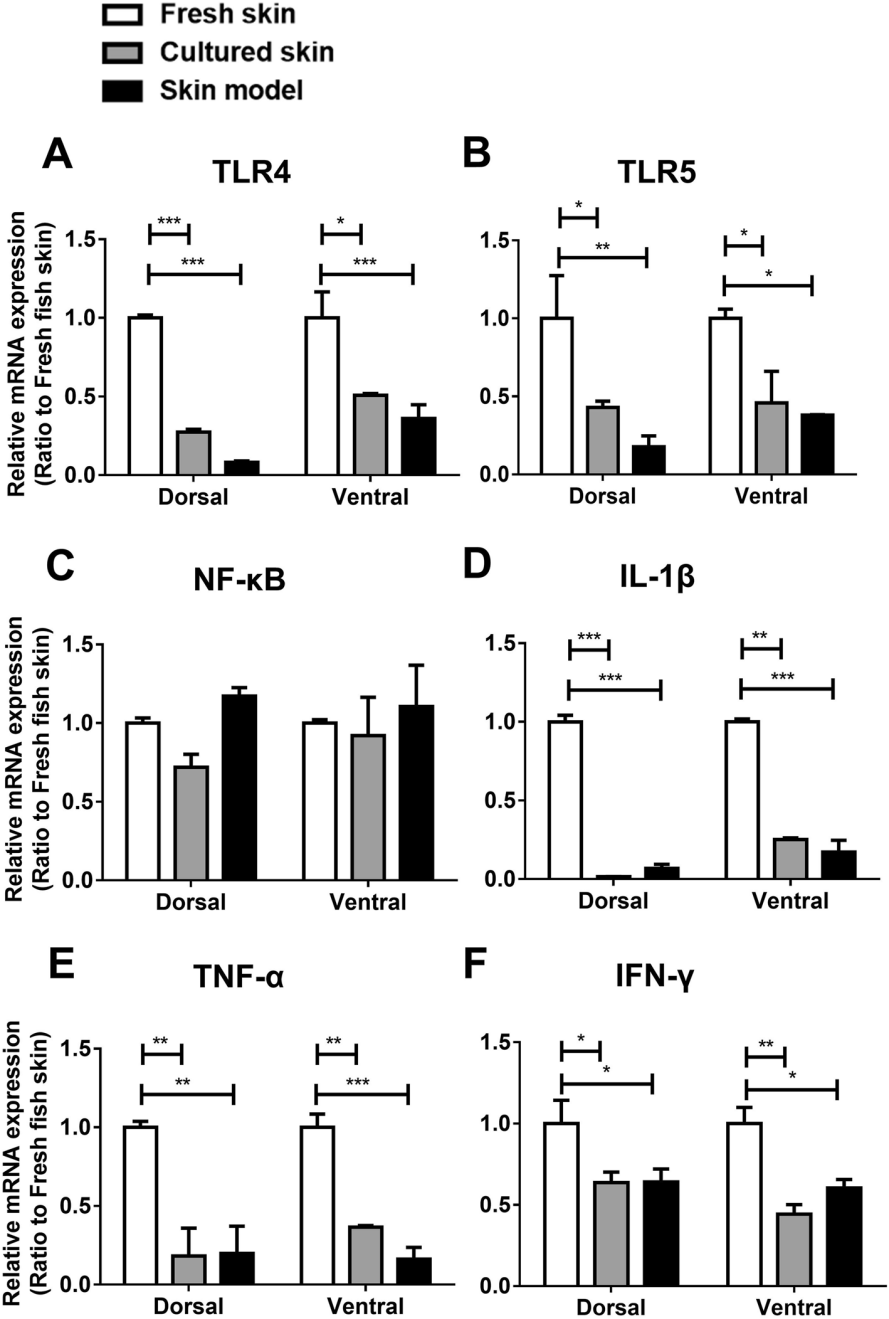
Immune marker mRNA expression in the skin model. The RNA from fresh skin, cultured skin, and skin model were extracted and followed by RT-qPCR for mRNA expression. The mRNA expression level of (**A**) TLR4, (**B**) TLR5, (**C**) NF-κB, (**D**) IL-1β, (**E**) TNF-α, (**F**) IFN-γ in cultured skin and skin model are relative to the expression of fresh skin. All data were shown as mean values (± SEM). Four fish and two replicates of tissue were used. Statistical significance was determined by using one-way ANOVA followed by Bonferroni post hoc test for comparison of expression between three conditions. *p ≤ 0.05 ***p ≤ 0.001; **p ≤ 0.01.

The innate immune system in teleost skin plays an essential role in response to pathogen surface molecules and in inducing inflammatory responses^14^. To investigate if the immune system was preserved and maintained in our skin model, we measured the expression level of pathogen recognition receptors TLR4 and TLR5, the intracellular signal transduction molecule NF-κB, and inflammatory cytokines IL-1β, TNF-α and IFN-γ using RT-qPCR in dorsal and ventral region and compared these levels to fresh skin or cultured skin (Fig. 5). In both dorsal and ventral regions, we found a significant decrease of TLR4 and TLR5 in the skin model and cultured skin compared to fresh skin (Fig. 5A,B). Similarly, a decrease of more than two fold in IL-1β and TNF-α was observed comparing fish skin and cultured skin to fresh skin (Fig. 5D,E). Lastly, a mild 1.6- to 2-fold decrease of IFN-γ expression was observed in the skin model and cultured skin compared to fresh skin (Fig. 5F). On the other hand, we did not find a significant change in NF-κB (Fig. 5C). These data suggest that the skin model could be responsive to innate immune signals, it has an overall lower level of expression in the innate immune system.

### AH elicited epidermal cell dissociation and barrier loss in the skin model

**Figure 6:**
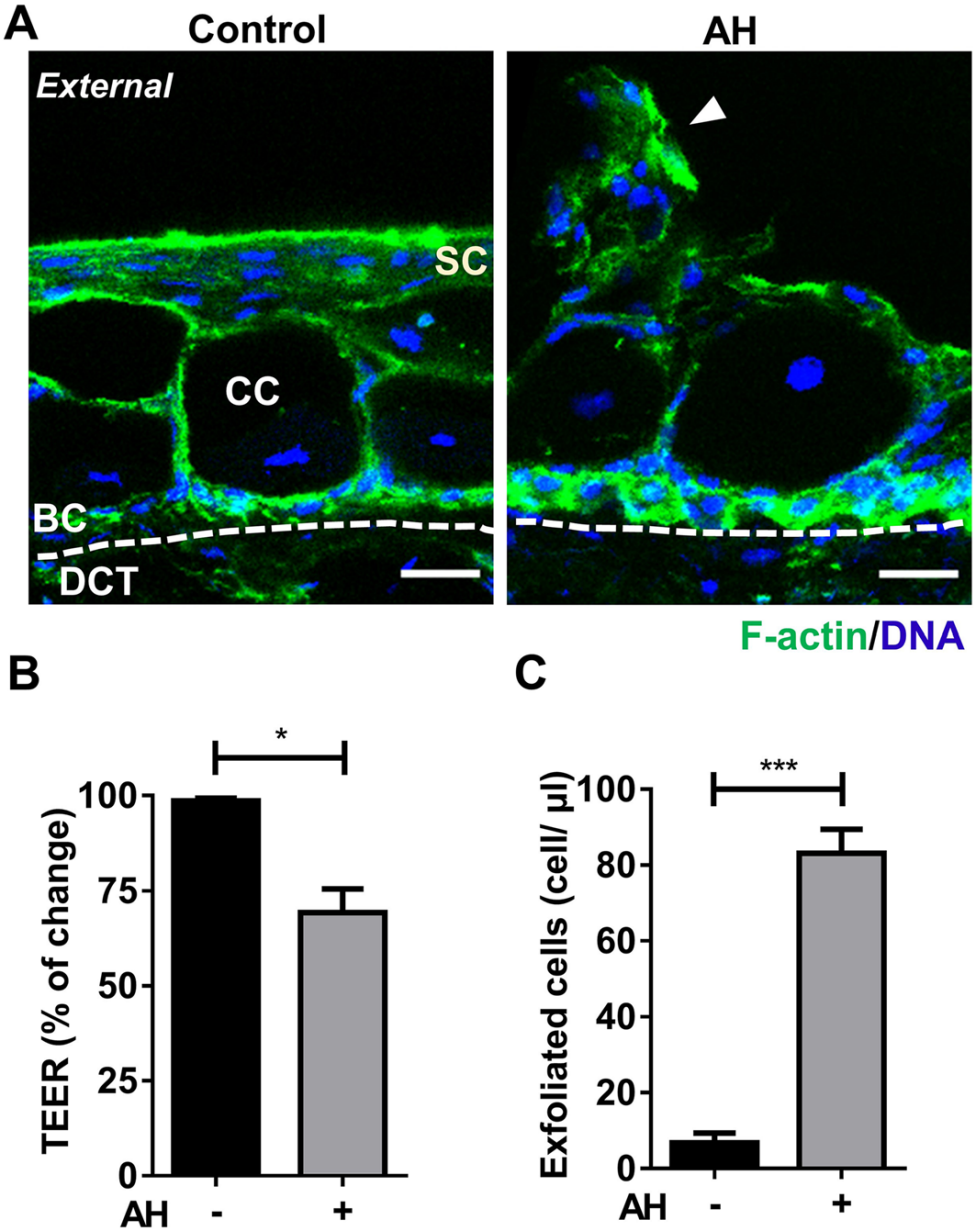
Epidermis cell damage in response to AH inoculation in the skin model. AH was inoculated into the ventral skin model and incubated for 6 h. (**A**) Tissue sections of skin model were stained for F-actin (green) and DNA stain (blue). Sections showing the superficial epithelial cells (SC), basal epithelial cells (BC), dense collagenous tissue (DCT), club cells (CC). The dissociated epithelial cells at surface epithelium was observed (arrowhead). Shown are representative images, Bar = 10 μm. (**B**) The transepithelial electrical resistance (TEER) was measured and percentage change was compared to TEER at 0 h. (**C**) Exfoliated cells in the apical chamber were counted using light microscopy. All data were shown as mean values (± SEM). Four fish and three replicates of tissue were used. Statistical significance was determined by using Student’s t-test. *p ≤ 0.05 ***p ≤ 0.001; **p ≤ 0.01.

Because previous studies reported that lesions of ventral skin region are common in various pathogen infections in catfish^31–33^, we used the ventral skin model to determine if it could be used as an infection model. We inoculated AH into the apical side of the ventral skin model for 6 h and investigated tissue integrity and the barrier function compared to no bacteria control. To test whether the tissue integrity changes upon AH inoculation, we examined the tissue morphology in the AH-inoculated skin model by F-actin staining and compared with no bacteria control (Fig. 6A). We found superficial epithelial cells were disassociated from surface epithelium compared to the control. To further test if AH inoculation exhibits barrier loss and tissue damage, we measured the TEER of the inoculated skin model and quantified the exfoliated cell number in the apical side compared to the no bacteria control. A significant 30% decrease in TEER (Fig. 6B) and tenfold increase in the exfoliated cell number (Fig. 6C) were observed in AH inoculated skin model compared to the no bacteria control. The results demonstrated that inoculation of AH could induce the epithelial dissociation and exfoliation along with barrier loss.

### The expression of innate immune markers is stimulated in AH-inoculated skin model

**Figure 7:**
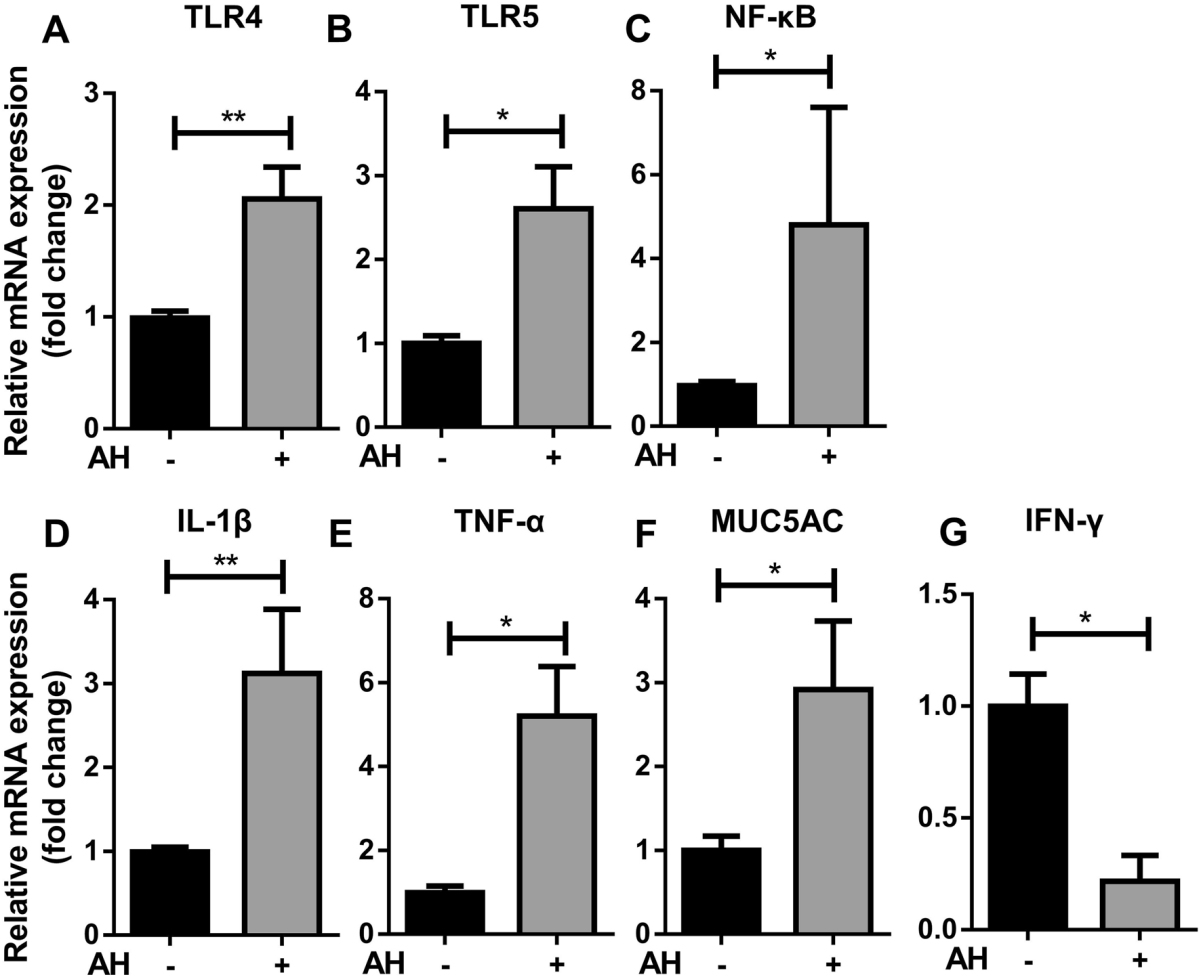
Effect of AH inoculation on innate immune marker expression in the skin model. The ventral skin model was inoculated with AH and incubated for 6 h. The RNA was extracted and followed by RT-qPCR for measuring expression. The expression of (**A**) TLR4, (**B**) TLR5, (**C**) NF-κB, (**D**) IL-1β, (**E**) TNF-α, (**F**) MUC5AC (**G**) IFN-γ in AH inoculated skin model were compared to expression in the no bacteria control assigned a value of 1. All data were shown as mean values (± SEM). Four fish and two replicates of tissue were used. Statistical significance was determined by using Student’s t-test. ***p ≤ 0.001; **p ≤ 0.01; *p ≤ 0.05.

The chained innate immune response from pathogen recognition to cytokine or antimicrobial secretion is the critical route to fight against infection^34^. To test if AH inoculation can elicit these responses, the expression of immune markers previously mentioned as well as mucous in AH-inoculated in the ventral skin model was measured by RT-qPCR. We found that all of the markers had significantly increased in AH-inoculated model compared to the control. The expression of TLR4 and TLR5 had a 2.3- and 2.7-fold increase respectively (Fig. 7A,B). The expression of NF-κB had a fivefold increase (Fig. 7C). The expression of two downstream pro-inflammatory cytokines, IL-1β and TNF-α, had a 3.3-fold and 5.7-fold increase, respectively (Fig. 7D,E). MUC5AC expression had a 3.6-fold increase (Fig. 7F). On the contrary, IFN-γ expression had a fivefold decreased (Fig. 7G). These data strongly suggested that innate immune response can be induced by AH inoculation in the skin model even though the basal level of expression is lower than in the fresh skin.

## Discussion

Little is known about bacterial infection in teleost skin. To better gain knowledge of teleost skin infection, we developed an ex vivo model. This culture method appears to be as stable as in vitro cell lines yet retains many of the properties of integrated in vivo models. This study established a tissue-based ex vivo teleost skin model that has not been explored before for studying skin infection. The skin model made of the teleost dorsal or ventral skin shows overall epidermal and connective tissue integrity. The expression of tight junction components and secretion of mucous resembles skin epidermis in vivo. Taking advantage of the slight amount of skin needed for the model, several sets of experimental conditions can be tested with a single fish, reducing the total animal sacrifice while eliminating the genetic difference between each fish. While the basal level of innate immune expression is relatively low compared to in vivo skin, elevated epithelial and immune responses to the pathogenic bacterium AH were observed. Overall, this skin model resembles in vivo teleost skin with or without infection. Thus, it can serve as an alternative or addition to the in vivo models currently used in pathogenesis research of teleost skin-related infections.

Epithelial polarity formation is critical for developing fully functional mucosal surfaces. Studies using rainbow trout have shown that culturing by separating apical and basal environments is necessary for the epithelial cell to form intercellular junctions leading to polarity^35^. Therefore, a skin model with polarized epithelial cells can be developed by creating distinct internal- and external-facing culturing environments. In rainbow trout RTgill-W1 cells cultured under transwell system has been shown to generate the polarity compared to non transwell system^36^. A mucosal epithelial cell model for many species, such as trout, tilapia, killifish, flounder, and sea bass has been made based on the concept of the transwell system^37^. In teleost fish, efforts towards polarizing epithelial have also been made. A previous study showed that a double seeded transwell system of gill epithelial cells exerts higher TEER and lower permeability than the single-seeded transwell system^38^. Our model improves on this concept having properties of polarity, including transepithelial resistance, and epidermal integrity with the function of mucous secretion. In contrast, cultured mucosal skin showed significant loss of upper epithelial and club cells.

We found that the epidermal layer of dorsal skin was noticeably thinner on day 15 compared to day 5 and 10 while the ventral skin was not observed (Supplementary Fig. S2). The observed change of cellular distribution but not TEER suggests that the skin model may have limited epidermal renewal capacity and a difference may therefore happen between the dorsal and ventral skin model. Two possible explanations can be discussed—the loss of environmental cues or the lack of proper growth factors. Studies have proved that polarization can be achieved by giving different environment cues on each side^39^. Berube et al. found that the lack of serum or hydration of the apical side polarizes human airway epithelial cells^40^. Dao Thi et al. found that successful hepatocyte polarization from stem cells can be achieved by removing serum from one side of the transwell system^41^. Both studies suggested the environmental cue is essential with feeding serum to the basal side only. The lack of proper growth factors can also change basal cell development. The dermis of fish skin is rich in the extracellular matrix (ECM), connecting epithelial cells to the underlying dermis. Studies have shown that epidermal growth factors can be stimulated by ECM and promote skin epidermal cell proliferation^42,43^. Basolateral changes of serum-free media and fish-specific hormone, such as epidermal growth factor and transforming growth factor^44^, may continuously stimulate the basal cell development and organization in the long-term culturing. One study showed that the average time for renewal of the teleost epithelial cells of skin epidermis is about 4 days^2^. It would be interesting to monitor the epithelial renewal of dorsal and ventral skin under the skin model settings in comparison to growth of isolated primary skin epithelial cells with or without the growth factors.

The existence of mucous cells and mucous secretion has long been an indication of fully developed mucosal surfaces. In scaleless fish such as catfish, mucous is often emphasized in defending pathogens and responding to surrounding chemical alteration^30^. Thus, morphometric studies have detailed the distribution and quantity of mucous cells in different species of catfish^45,46^. Very few studies have conclusive findings on the number and the distribution of mucous cells in *P. hypophthalmus*. Our findings have shown that the distribution and quantity of GC in both fresh skin and our skin model were similar. The mucin expression is also similar. Consistent with the data, mucous secreted from GC were observed in both fresh skin and the skin model located at the superficial epithelial cell surface (Supplementary Fig. S1). The images indicate the integrity and functionality of a fully grown skin mucosal epithelium. Club cells, which are the second most common cell type in the epidermis, have also been suggested to contain and potentially secret proteinaceous mucous^47,48^. Two types of mucous-secreting cells are shown here, potentially secreting a mixture of mucous. It would be interesting to examine the composition of the secreted mucous in our model and to compare the physiological existence of certain glycosylated or un-glycosylated mucous with fresh skin.

The expression of immune markers is an indicator of the immune response to various pathogenic bacteria. The established cytokine secretion pathway from surface TLRs down to ILs is often used to validate the established pathogen defense pathways^6^. TLR4/5 has been identified in catfish to function as acute innate immune markers and shows an increased level in response to infection with pathogenic bacteria^6,49^. TNF-α and IL-1β constitute classical pro-inflammatory cytokine, which can clear the pathogens at the acute phase of an immune response whereas IFN-γ is an effector of cellular responses which can enhance the immune response against pathogens. Interestingly in our model cultured in media with antibiotics, we noticed a deceased expression of TLRs, IL-1β, TNF-α and IFN-γ compared to fresh skin. We reason that the lack of mucosal microbiome may be the cause of this decreased expression.

Numerous studies have found an immunostimulant function of fish mucosal microbiome^14,50^. Also, similar evidence showed in gnotobiotic zebrafish that in the absence of a microbiome, they have reduced immune gene expression compared to the one exposed to microbiome^51^. The lack of microbiome may explain the low immune activation in our skin model because it is cultured in media with antibiotics. However, this could be readily tested by adding back bacteria in antibiotic-free media. Furthermore, it could be adapted to define the types of organisms providing this stimulation. The absence of the microbiome can represent the gnotobiotic fish skin where immunostimulation is lacking. However, the microbiome can also serve as a buffer to reduce skin mucosal immune overexpression and maintain stability when encountering the pathogen^14^. Li et al. showed that, even with partial mucous removal, the innate immune marker expression maintains the same level during 2–12 h after infection of AH using the blue catfish^52^. This implies an immune-suppression role of the mucous microbiome in fish infection, which explains the immediate upregulation of TLR4 and TLR5 along with IL-1β and TNF-α in our skin model inoculated with a low number of AH. The loss of the mucosal microbiome may contribute to the decreased buffering and, therefore, a robust immune response to infections. However, this does not seem to apply to IFN-γ, in which the expression level was decreased under AH infection. A similar result of decreased IFN-γ regulation has been found under in vivo AH infection of blue catfish, indicating a less induced immune marker for AH infection^52^. Overall, the microbiome potentially plays a role in modulating skin immune response.

Although the skin model can resemble the in vivo innate immune functionality of skin epithelial in our experiment system without the microbiome, there are still limitations to consider. One limitation is the reduction of in vivo level of immune cells and their activation. In general, epithelial cells of stressed fish can release cytokines to activate and direct immune cells to the targeted tissue^3^. High numbers of activated macrophages in the skin and increased T cell activation are then responsible for pathogen recognition and eventually clearance^53^. In our skin model, however, immune cells are not expected to be retained after long-term culture. Therefore, many cytokines secreted by immune cells in our skin model, such as IL-17 by T-cells, would not be able to achieve in vivo level. Another limitation is the fish endocrine system response. Maintenance of fish skin homeostasis relies on immune and endocrine balance. Small et al. showed that cortisol released by the fish gland endocrine system could suppress the inflammation to protect tissues from cytotoxic damage^54^, while Pagniello et al. indicated cortisol induced the proliferation of macrophages in rainbow trout cell line^55^.

Our teleost skin model resembles in vivo fresh skin with tissue morphology and characteristics preserved. The whole tissue without chemical or physical modification and damage has been proven and used to mimic in vivo organ or tissue in a physiologically identical manner. In a sheep study, a 3D skin ex vivo model made from biopsies has been developed in the same manner and used for investigating anaerobic bacterial infections and the host immune response under the skin^56^. Another study developed an ex vivo human peritoneal tissue model to explore mesh-tissue integration using explants^22^. In these investigations, the model can be maintained for two weeks, ideal for applicable infectious disease observation. However, the limited epithelial renewal found in these models leaves the need for improvements to perform long-term experiments. The fish skin model system developed in this study can last for at least 18 days, serving as a model for short-term infection or other physiological studies. A further study exploring the extended growth and maintenance of this model is needed.

Taken together, this skin model has shown the similar epidermal property and functionality of fresh skin for weeks. The pathogen challenge can induce the activation of an innate immune response responding to pathogen challenge. To our best knowledge, this explant-based ex vivo skin model has not been explored before. Thus, this model can potentially serve as a platform for studying fish skin infection and its control and prevention prospectively.

## Materials and methods

### Animal husbandry

*Pangasius hypophthalmus* were obtained from a local aquarium vendor. Around twenty fish were housed in a 300 L tank at 25 °C and held constant photoperiod (12 h light; 12 h dark) in the aquaculture room at the Department of Marine Biotechnology and Resources. Fish skin tissue was prepared from observationally healthy fish with an average weight of 35 g and ten months old. This study was carried out in compliance with the ARRIVE guidelines. All the animal experiments were approved by the Institutional Animal Care and Use Committee (I.A.C.U.C.) at National Sun Yat-sen University under protocol No. IACUC-10834.

### Skin tissue collection

Fish were anaesthetized by rapid chilling followed by cervical transection. The experiment was conducted in accordance with AVMA Guidelines for the Euthanasia of Animals. The fish skin tissue was then removed from the fish by scalpel and immediately immersed into cold L-15 medium (SIGMA, U.S.A.) supplemented with 10% fetal bovine serum, 2% gentamycin solution (SIGMA, U.S.A), 1× antibiotic–antimycotic (Biowest, U.S.A.).

### Fabrication of model

The skin tissue was separated into the ventral and dorsal regions based on the lateral line. Skin tissues of dorsal and ventral region were collected from dorsal fin and pelvic fin to lateral line, respectively. After cutting into squares of approximately 10 × 10 mm, skin tissue was fixed in the upper plastic crown using a fine rubber band and mounted with the lower plastic crown (Fig. 1B). The tissue, along with the crown, was gently submerged in the culture medium in a 24-well culture plate. In cultured skin, the skin was submerged in the culture medium in a 24-well culture plate. The plate was cultured in a CO_2_-free incubator at 25 °C. The culture medium was changed every two days until further experiments. The media was replaced with non-antibiotic media 24 h before the infection experiment.

### Bacteria cultivation and infection

*Aeromonas hydrophila* (AH) was purchased from B.C.R.C. (No. 16704), Taiwan. The bacterium was cultured on the starch ampicillin agar plate (HiMedia, India) in a CO_2_-free incubator at 30 °C for 15–18 h before inoculation. The skin model cultured for 5 days were inoculated with AH at 10^3^ CFU/ml in apical media and incubated at 25 °C for 6 h.

### Histological examination of the skin

Tissue samples were rinsed with 1× phosphate-buffered saline (1× PBS) and fixed in 4% paraformaldehyde for 24 h. Samples were then undergone gradual dehydration with sucrose and embedded in 20% gelatin in 1× PBS and stored at – 80 °C. Twenty-micron thick sections were cut using a cryo-microtome (MICROM HM550, Thermo, U.S.A.), and sections were stained with either Giemsa stain (SIGMA, U.S.A.) for tissue integrity or Alcian Blue stain (ScyTek, U.S.A.) for labeling goblet cells. All the prepared slides were stored at 4 °C for later light microscopy examination.

### Total RNA extraction and cDNA preparation

RNA from the skin tissue of *P. hypophthalmus* was extracted with the TriPure Isolation Reagent (Roche, Mannheim, Germany) following the manufacturer's instructions. The RNA pellet was dissolved in nuclease-free water. Extracted RNA samples were stored at − 80 °C. The reverse transcription was then performed using M-MLV Reverse transcriptase (Promega, U.S.A.) to synthesize cDNA following the manufacturer’s instructions. The successful construction of cDNA library was determined by 1.5% agarose gels containing the Safeview DNA stain (GeneMark, Taiwan).

### Real-time qPCR

The quantitative real-time PCR reaction was performed using the GoTaq qPCR Master Mix (Promega, U.S.A.) on a CFX96 real-time PCR Detection System (Bio-Rad, U.S.A.). The primers used for RT-qPCR are listed in Supplementary Table S1. The thermal cycling profile consisted of an initial denaturation at 95 °C for 2 min, followed by 40 cycles of denaturation at 95 °C for 3 s, an appropriate annealing/extension temperature at 60℃, for 30 s. The comparative Ct (ΔΔCt) method was used to evaluate the expression of candidate genes^57^. Basically, transcript abundance for each gene was normalized using housekeeping gene elongation factor 1-alpha (EF1-α). The expression level of each gene was calculated by 2^−ΔΔCt^. All data were given in terms of relative mRNA expressed as means ± SEM. Four independent experiments of fish with 2–3 technical replicates were performed. The Ct of each replicate was reading three times for accuracy.

### Fluorescence staining

Sections were rinsed with warm 1X PBS to remove gelatin and incubated with 0.2% Triton X-100 in 1× PBS for 1 h at room temperature. The sections were then incubated with Phalloidin-Alexa Flour 488 (Invitrogen, U.S.A.) for 1 h for F-actin staining. After rinsed in 1XPBS, all the sections were then incubated with Hoechst33342 (Sigma, U.S.A.) for 20 min for nuclear DNA staining. The sections were mounted and stored in 4 °C. The samples were imaged using the Leica TCS SP5 II confocal microscope (Leica, Germany) or Leica DM 6000B light microscope with a SPOT Idea 5 M.P. Scientific Digital Camera System (Diagnostic Instruments Inc., Sterling Heights, U.S.A.).

### Statistical analysis

Statistical significance was assessed using the Student's two-tail t-test or one-way analysis of variance (ANOVA) followed by Bonferroni multiple comparisons depends on comparable properties. All the data were confirmed to fit into Gaussian distribution by Shapiro–Wilk test for normality. Homogeneity of variance was confirmed using Bartlett's test and F-test. For t-test, if heterogeneity of variance was found, a Welsh’s test was performed to reassure the statistical analysis. All the analyses were performed using GraphPad Prism8 software (https://www.graphpad.com/).

## Supplementary Information

Supplementary Information.
