# Supplementary material for: Establishment of a striped catfish skin explant model for studying the skin response in Aeromonas hydrophila infections
Source: Sci Rep. 2021 Sep 24;11:19057. doi: 10.1038/s41598-021-98583-8 (PMC8463585; doi:10.1038/s41598-021-98583-8)
Supplement: Supplementary file 1 — Supplementary Information. [file 41598_2021_98583_MOESM1_ESM.docx]

**Supplementary information**

**Establishment of a striped catfish skin explant model for studying the skin response in *Aeromonas hydrophila* infections**

Ru-Fang Siao ^1,+^, Chia-Hsuan Lin ^1,+^, Li-Hsuan Chen ^1,+^, Liang-Chun Wang ^1,+,*^

**1**Department of Marine Biotechnology and Resources, National Sun Yat-Sen University, Kaohsiung, Taiwan

^+^These authors contributed equally to this work

*Corresponding author.

*E-mail*: [marknjoy@g-mail.nsysu.edu.tw](mailto:marknjoy@g-mail.nsysu.edu.tw)


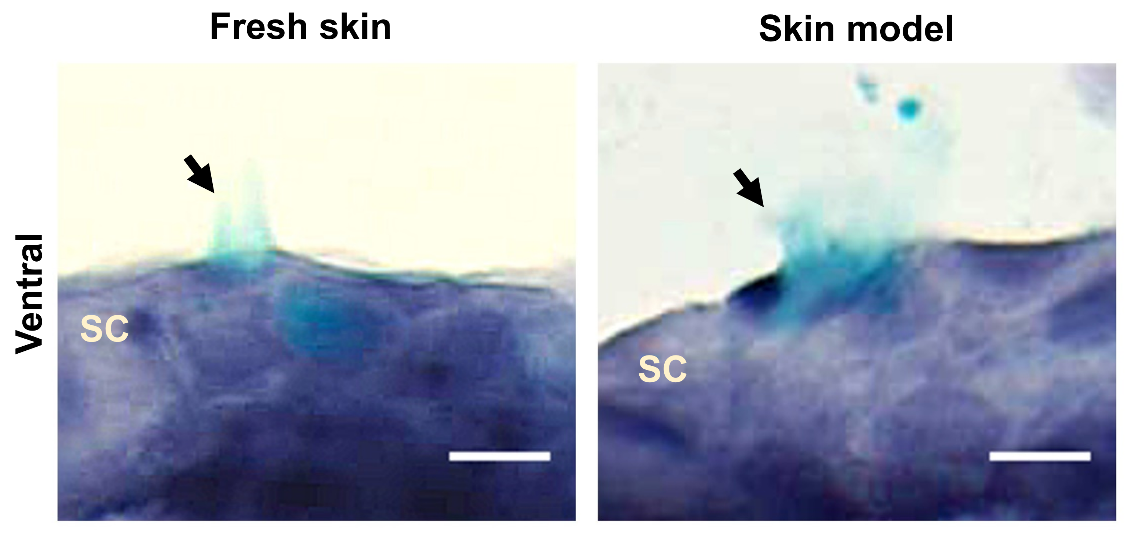


**Supplementary Figure. S1** **Mucous secretion at the surface of the skin model.** Tissue sections from fresh skin and skin model of the ventral region were stained with Alcian blue-hematoxylin stain. Arrows point the secreted mucous from mucous goblet cell located at superficial epithelial cells (SC) surface. Shown are representative images from 4 independent experiments, Bar = 10μm.


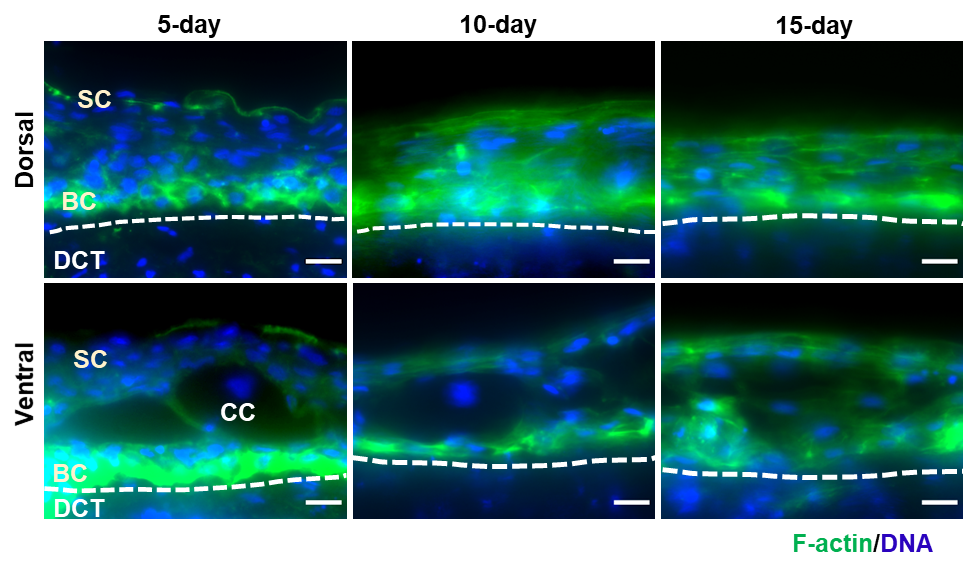


**Supplementary Figure. S2 Tissue morphology in the skin model cultured for 5, 10, and 15 days.** Cryosections of 5-day, 10-day, and 15-day skin models were stained for F-actin (green) and DNA (blue). Sections showing the superficial epithelial cells (SC), basal epithelial cells (BC), dense collagenous tissue (DCT), club cells (CC). Shown are representative images from 4 independent experiments, Bar = 10μm.

**Supplementary Table. S1** **Primers sequences used in qPCR**

| Target gene | Primer | Primer sequence (5’→3’) | Product length (bp) | Accession no. |
| --- | --- | --- | --- | --- |
| EF-1α | Forward  Reverse | AGGACATCCGTCGTGGTAAC  TCAGGATGATGACCTGAGCA | 90 | XM_026930196.2 |
| ZO-1 | Forward  Reverse | AAAATCCTCCGACTCCTCGT  CAGTTCGCTCCAACCAAGTAG | 90 | XM_034299525.1 |
| Claudin-1 | Forward  Reverse | GATGACGTGGAGCTCAAAAAC  AGCAACGAAAGCACAGACAC | 81 | XM_034303602.1 |
| Occludin-1 | Forward  Reverse | GTTGAAAAGCCGAACGAGAG  ATCGGAGGGAACTCACTGTC | 90 | XM_034299259.1 |
| MUC5AC | Forward  Reverse | GTGTTTCAATACTACGACACGG  AAAGGGCAACACGTTCACTC | 158 | XM_034297821.1 |
| TLR4 | Forward  Reverse | GCAAGGCAATGCTAACATCAT  GAAGACCAAACACCTTCTTTGAC | 74 | XM_026932800.2 |
| TLR5 | Forward  Reverse | TCATCTCGGTTCTGTTGATCC  AAGCGGTTTCCAGACAACTC | 69 | XM_034310181.1 |
| NF-κB | Forward  Reverse | TGGGCTATTCCTTCACTTCAC  TGACATACCCACTCCGTTACTG | 72 | XM_026937724.2 |
| IL-1β | Forward  Reverse | CAGTGCAAATGTGTCAGCAG  GGTCTCATCATGAAGCGTGA | 62 | XM_034312378.1 |
| TNF-α | Forward  Reverse | GCAGACCAGTCTTTCGCTTC  GGCCATTACGGAGAATCTTG | 67 | XM_026942329.2 |
| IFN-γ | Forward  Reverse | TCCCAACCCTGCCAAATTGT  GCCTCATTCTCCATCCAGGT | 150 | XM_026923590.2 |
